# Cercarial emergence pattern of *Schistosoma haematobium* from Libreville, Gabon

**DOI:** 10.1051/parasite/2014004

**Published:** 2014-02-10

**Authors:** Rodrigue Mintsa-Nguéma, Hélène Moné, Moudachirou Ibikounlé, Krystina Mengué-Ngou-Milama, Maryvonne Kombila, Gabriel Mouahid

**Affiliations:** 1 Institut de Recherche en Écologie Tropicale (IRET), CENAREST-Gabon BP 13354 Libreville Gabon; 2 Université Perpignan Via Domitia, Écologie et Évolution des Interactions, UMR 5244 66860 Perpignan France; 3 CNRS, Écologie et Évolution des Interactions, UMR 5244 66860 Perpignan France; 4 Département de Zoologie et Génétique, Faculté des Sciences et Techniques, Université d’Abomey-Calavi 01BP526 Cotonou Bénin; 5 Service de Parasitologie-Mycologie-Médecine Tropicale, Faculté de Médecine de Tours, CHRU Bretonneau 37044 Tours France; 6 Département de Parasitologie-Mycologie, Université des Sciences de la Santé de Libreville BP 4009 Libreville Gabon

**Keywords:** Bilharziasis, Gabon, Transmission, *Schistosoma haematobium*, Cercarial emission, Chronobiology

## Abstract

Although schistosomiasis has been a public health issue in Gabon for nearly a century, little is known about its current transmission dynamics. We analyzed the chronobiology of 137 cercarial emission profiles of *Schistosoma haematobium* from Libreville, the capital of Gabon, located in an open area for schistosomiasis. We found that 88% of the cercariae were shed between 11 a.m. and 3 p.m. and that the average pattern was of circadian type, with the average peak at 1 p.m., and representing 27% of the total number of cercariae of the day. The rhythms of emergence may be associated with environmental pressures on the parasite, especially those related to their definitive hosts.

## Abstract (fr)

Bien que la schistosomose soit un problème de santé publique au Gabon depuis près d’un siècle, la dynamique actuelle de sa transmission est peu connue. Nous avons analysé la chronobiologie de 137 profils d’émission cercarienne de *Schistosoma haematobium* de Libreville, la capitale du Gabon, située dans une zone géographique ouverte pour la schistosomose. Nous avons trouvé que 88 % des cercaires étaient émises entre 11 heures et 15 heures et que le profil moyen était de type circadien, avec un pic moyen situé à 13 heures et représentant 27 % du nombre total de cercaires journalier. Les rythmes d’émergence sont associés aux pressions environnementales sur le parasite, particulièrement celles relatives aux hôtes définitifs.

## Introduction

Schistosomiasis is a public health problem throughout the world. At present, 779 million individuals are at risk of infection and more than 207 million people are infected, with 97% of the latter in Africa [20].

The first Bilharziasis reported in Gabon was a rectal schistosomiasis, called Schistosomum *haematobium*, which occurred in Libreville, the capital of Gabon, in 1923 [2]. It was later demonstrated that the agent responsible for this schistosomiasis was *Schistosoma guineensis*. Bladder schistosomiasis due to *S. haematobium* was described more than 40 years later [5] in the village of Moukoro, in the Ndendé district in the south of Gabon; this schistosomiasis was introduced from N’Kayi (formerly Jacob), located on the Congolese border of Gabon. The prevalence of *S. haematobium* infection in the population at that time was 50%. Its expansion into the rest of the country likely occurred quickly, from the south to the northwest, following the rivers and roads within the country. Indeed, *S. haematobium* was detected in 1967 near the Tchibanga Nyanga area in the southwest of Gabon [5] and in 1969 in the Palmhévéas region, near Lambaréné (middle Ogowe) [5]. In 1974, an epidemic was reported in Ekouk, North of Palmhévéas, near Libreville [9], with populations living along the road connecting the Niari Valley in Congo to Libreville being the most vulnerable to infection. In 1981, *S. haematobium* left the main road and spread in different directions, especially in the west of the country, in the province of Ogooué Maritime (Port-Gentil) and in the southeast, in the province of Upper Ogooué (Moanda). In 1982, seven of the nine provinces of Gabon were affected by urinary schistosomiasis, with the largest foci in Libreville, Koulamoutou in Ogooué Lolo Province, Bibora in Nyanga Province, and Palmhévéas in Moyen Ogooué Province. The prevalence of infection was generally higher than that of *S. guineensis* [7, 10]. Thus, at present two schistosome species adapted to humans coexist in Gabon, which has led to research on the possible existence of hybrids [1, 10, 18].

*Schistosoma haematobium* is a species very sensitive to light [16, 17]. It was shown, in Côte d’Ivoire, that cercariae located in shady transmission sites situated in forest areas were emitted earlier (11 a.m.) than those located in transmission sites receiving direct sunlight, such as savanna areas (1:30 p.m.) [15]. The progression of *S. haematobium* from the South of Gabon to Libreville follows a vegetal axis represented mainly by savannas, degraded forests, and mangroves, resulting in conditions of strong light in potential sites of transmission, whereas the rest of the country (85%) is covered by rainforests [8]. The purpose of this study was to analyze *S. haematobium* cercarial emissions from transmission sites located in Libreville, where the forest has disappeared.

## Material and methods

### Biological material

The strain of *Schistosoma haematobium* originated from Melen (09°30′59″ E, 00°23′46″ N) in Libreville city, Gabon [10]. The F_0_ generation was extracted from positive urine samples of nine patients and transported to France in experimentally infected *Bulinus globosus* from Toho-Todougba, Benin (2°11′21″ E, 6°23′52″ N). The strain was then maintained in jirds (*Meriones unguiculatus*) as definitive hosts. We used the F_1_ generation of the parasite for the cercarial emission pattern analyses.

### Infection and maintenance of snails

The snails were exposed individually to five miracidia of the F_1_ generation, a dose which is usually used in laboratories because it allows one to obtain an optimal percentage of snail infection [12, 13, 14, 19]. The exposed snails were maintained in aquaria containing well water in a temperature-controlled room at 24–25 °C. They were fed ad libitum with fresh lettuce. The photoperiod was balanced, with 12 h of light (from 6:00 a.m. to 6:00 p.m.) and 12 h of darkness (from 6:00 p.m. to 6:00 a.m.), with a simulated dawn (from 5:30 a.m. to 6:00 a.m.) and simulated dusk (from 5:30 p.m. to 6:00 p.m.). At the end of the prepatent period (the time between exposure of the snails to the miracidia and the first cercarial emission), the snails were placed individually in plastic cups containing 150 mL of well water, in a balanced photoperiod light: dark of 12:12.

### Cercarial emission patterns

Every hour, starting at 7:00 a.m. (corresponding to the cercariae emitted from 6 a.m. to 7 a.m.), each infected snail was transferred individually to another glass containing the same volume of well water at the same temperature (24–25 °C). The water samples containing the cercariae were filtered through polyamide Nitrile filters (25-μm mesh sieve). The cercariae retained on the filter were stained with Lugol’s solution and counted under a dissecting microscope. To strengthen each profile, we retained only the emission profiles for which the total daily cercarial production was ≥50 (range: 53–837 cercariae per day). In total, 14 snails (G1 to G14) were analyzed and followed for 5–14 consecutive days, yielding a total of 137 cercarial emission profiles (Table 1).

**Table 1.** Variation of the position of the cercarial emission peak for each individual snail.

Snail	NCEP	Hour					
		11 a.m.	12 a.m.	1 p.m.	2 p.m.	3 p.m.	4 p.m.
G1	9	1	6	1	1		
G2	8		5	3			
G3	8		4	4			
G4	9	5	4				
G5	9		2	7			
G6	13	1	4	5	2		1
G7	13		6	3	4		
G8	14		5	9			
G9	5		3	2			
G10	9	1	2	2	2	2	
G11	6	1	1	4			
G12	13	1	3	3	3	3	
G13	7		1	4	2		
G14	13		4	5	4		1
Total	137	10	50	52	18	5	2

NCEP: Number of cercarial emission profiles.

### Ethics statement

The laboratory has received the permit No. A 66040 for experiments on animals, from both the French Ministère de l’Agriculture et de la Pêche and the French Ministère de l’Education Nationale de la Recherche et de la Technologie (Décret n° 87-848 du 19 octobre 1987). The housing, breeding, and care of the rodents followed the ethical requirements of our country. Animal experimentation followed the guidelines of the French CNRS. The different protocols used in this study have been validated by the French Veterinary Agency.

## Results

**Figure 1.**
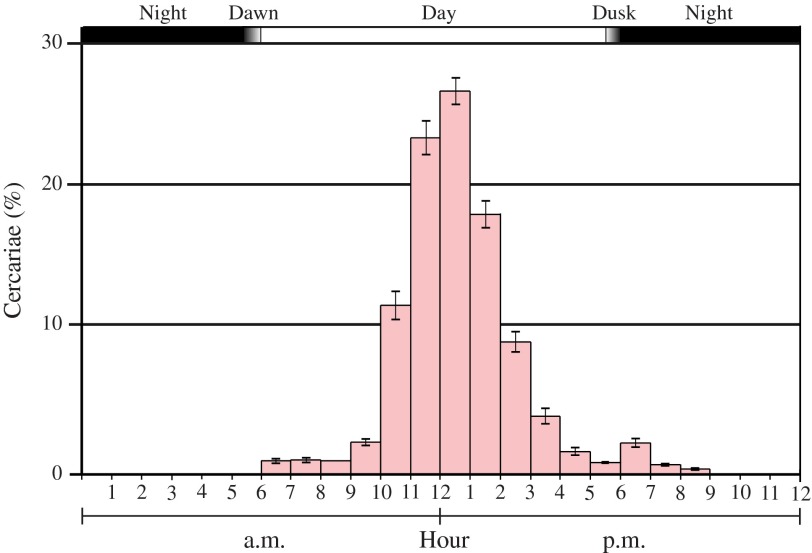
Average percentage of cercariae of *Schistosoma haematobium* (Libreville, Gabon) emitted every hour from *B. globosus* (Benin), each exposed to five miracidia. Bars represent standard errors.

The histogram representing the profile of the average daily cercarial emissions of Gabonese *S. haematobium* from *B. globosus* snails from Benin is presented in Figure 1. The emergence of cercariae tentatively began at 7 a.m. (i.e. at the beginning of the photophase), increased gradually from 10 a.m. to its peak at 1 p.m., and decreased gradually before stopping completely at 8 p.m. (i.e. 2 h after the beginning of the scotophase). The majority of the cercarial emissions (88%) occurred between 11 a.m. and 3 p.m. The average emission peak occurred at 1 p.m. and represented 26.3% of the total daily cercarial production.

The variation of the position of the cercarial emission peaks is presented in Table 1. The 137 peaks were situated between 11 a.m. and 4 p.m.: 10 peaked at 11 a.m., 50 at 12 a.m., 52 at 1 p.m., 18 at 2 p.m., 5 at 3 p.m., and 2 at 4 p.m. For each individual snail and for each profile, only one peak was observed (circadian rhythm); none of the snails had all its peaks situated at the same hour. Each snail had 2, 3, 4, 5, or 6 peak positions according to its profiles. For example, the snail G1 had four peak positions (one peak at 11 a.m., six at 12 a.m., one at 1 p.m., and one at 2 p.m.) and the snail G4 had two peak positions (five peaks at 11 a.m. and four at 12 a.m.). However, for each snail, two peak positions were over-represented, 12 a.m. and 1 p.m. Five snails (G2, G3, G5, G8, and G9) had 100% of their peaks situated either at 12 a.m. or at 1 p.m.; the other snails had from 44.4 to 83.3% of their peaks situated either at 12 a.m. or at 1 p.m.

## Discussion

Our results on the chronobiology of *S. haematobium* cercarial emission in Libreville showed that the rhythm of emergence was of a circadian type, with 88% of the cercariae emitted between 11 a.m. and 3 p.m. and an average emergence peak at 1 p.m., representing 27% of the cercariae emitted daily. The average emergence peak is similar to that obtained in Ivory Coast for a strain of *S. haematobium* located in the vegetal zone called wooded savanna corresponding to the sub-Sudanian climate [15]. Historically, the course of migration of our strain of *S. haematobium* was from the south to northwestern Gabon, corresponding, for the most part, to savanna and mangrove areas [8].

The cercarial emergence behavior is genetically supported [23], with this behavior remaining unchanged when the schistosome is subject to different environmental pressures at the level of its intermediate snail host. For example, the emission profile of *S. haematobium* from the snail *B. truncatus* does not change when the snail is also infected with another species of schistosome, *Schistosoma bovis* [12], and the emission profile of *Schistosoma mansoni* from the snail *Biomphalaria glabrata* does not change when the snail is infected with another fluke, *Ribeiroia marini* [24]. Moreover, the emission patterns of two strains (early and late) of *S. mansoni* remain unchanged when both parasites are present simultaneously in *B. glabrata* [25], and the emission profile of *S. mansoni* remains unchanged when infected *B. glabrata* is subject to the presence of a competitor snail, *Marisa cornuarietis* [4].

From an evolutionary point of view, the diversity of the rhythms of emergence is usually well correlated with the diversity of behavior of the definitive host species [3]. In the region of Libreville, the circadian rhythm of emissions, with a peak at 1 p.m., can be explained by human contact with water, which is essentially related to bathing activities during the hottest hours of the day, between 11 a.m. and 4 p.m. In other areas, the practice of activities other than swimming, such as artisan fishing, can result in a change in the cercarial emission pattern to an ultradian pattern, with a primary peak at midday but secondary peaks at dawn and dusk, when fishermen are in contact with water, as has been shown in *S. mansoni* in Benin [6]. Some strains of *S. mansoni* have adapted to the presence of definitive hosts other than humans, such as the rodent, *Rattus rattus*, with the cercarial emission peak becoming later, as in Guadeloupe [22], or strictly nocturnal, as in Oman [11]. This has also been shown for the species *Schistosoma japonicum*, with cercarial emergence peaks occurring later or becoming nocturnal, due to the reservoir hosts, particularly rodents, used by this species [21]. Indeed, the involvement of murine reservoir hosts in the transmission dynamics has led to intraspecific variability in cercarial emission, with distinct chronobiological races corresponding to sites in which humans and/or reservoir hosts are the definitive hosts. The cercarial emission peaks at 1 p.m. in the open area of Libreville may be related to different behaviors of the definitive host, man, in different types of landscape, with the shady forest requiring activities be performed earlier in the day to compensate for the lack of light, whereas activities are performed later in open areas such as savannas and mangroves.
